# Digital biomarkers of mood disorders and symptom change

**DOI:** 10.1038/s41746-019-0078-0

**Published:** 2019-02-01

**Authors:** Nicholas C. Jacobson, Hilary Weingarden, Sabine Wilhelm

**Affiliations:** 1 Massachusetts General Hospital, Boston, MA USA; 2 Harvard Medical School, Boston, MA USA; 3 The Pennsylvania State University, University Park, PA USA

**Keywords:** Diagnostic markers, Human behaviour, Predictive markers

## Abstract

Current approaches to psychiatric assessment are resource-intensive, requiring time-consuming evaluation by a trained clinician. Development of digital biomarkers holds promise for enabling scalable, time-sensitive, and cost-effective assessment of both psychiatric diagnosis and symptom change. The present study aimed to identify robust digital biomarkers of diagnostic status and changes in symptom severity over ~2 weeks, through re-analysis of public-use actigraphy data collected in patients with major depressive or bipolar disorder and healthy controls. Results suggest that participants’ diagnostic group status (i.e., mood disorder, control) can be predicted with a high degree of accuracy (predicted correctly 89% of the time, kappa = 0.773), using features extracted from actigraphy data alone. Results also suggest that actigraphy data can be used to predict symptom change across ~2 weeks (*r* = 0.782, *p* = 1.04e-05). Through inclusion of digital biomarkers in our statistical model, which are generalizable to new samples, the results may be replicated by other research groups in order to validate and extend this work.

## Introduction

Mood disorders (i.e., major depressive disorder [MDD], bipolar I, bipolar II) occur in 12% of the population in their lifetime, resulting in substantial functional and economic burden.^1^ To reduce burden, it is critical to diagnose and monitor mood disorders using widely accessible methods, which enable timely detection of clinical deterioration. However, standard clinical assessments require time-consuming and resource-heavy evaluation by a trained specialist.

Use of passive digital biomarkers offers an alternative approach to assess mood disorders that is scalable, unobtrusive, time-sensitive, and cost-effective. Movement data from actigraphs (which measure gross motor activity from a sensor typically worn on the wrist, capturing both activity and rest) may be especially useful for detecting MDD or bipolar disorders, because these disorders are characterized by notable increases (i.e., in bipolar disorder) or decreases (i.e., in MDD) in goal-directed behavior, energy level, and movement, in addition to disruption in sleep— behavioral shifts which are likely to be captured via actigraph.

A small base of research has explored using actigraphy data for mood disorder diagnosis.^2,3^ One investigation used daytime and nighttime movement data from actigraphs worn on wrists of patients with primary bipolar disorder or MDD and controls to classify participants’ diagnostic group (kappa = 0.443, accuracy = 72.7%), using support vector machines (a machine-learning algorithm, which attempts to create the greatest difference between groups on dimensional hyperplanes).^4^ However, the authors utilized features that were context-dependent (data were directly referenced to time within study, e.g., movement on minute 1 within the study), rather than time-relative (e.g., the consistency of movement between 1 day to the next on average within the sample). This limits the generalizability of their methods to new samples, because linking data to absolute time does not allow for generalization to shorter or larger timespans. Indeed, most such studies to date yield only modest results^3^ and oftentimes are not replicable. Moreover, while most biomarker research has focused on detecting diagnosis, very few studies have aimed to detect symptom change.^3^ Developing unobtrusive, timely methods to detect symptom change has potential to enable just-in-time interventions to prevent clinical deterioration as a next step.

To extend current research, we re-analyzed public-use data^4^ using novel methods, with the aim of identifying digital biomarkers of MDD and bipolar disorder diagnostic status and changes in symptom severity over ~2 weeks. We sought to use methods that are generalizable to new samples, so that results may be replicated by other research groups.

## Results

**Fig. 1:**
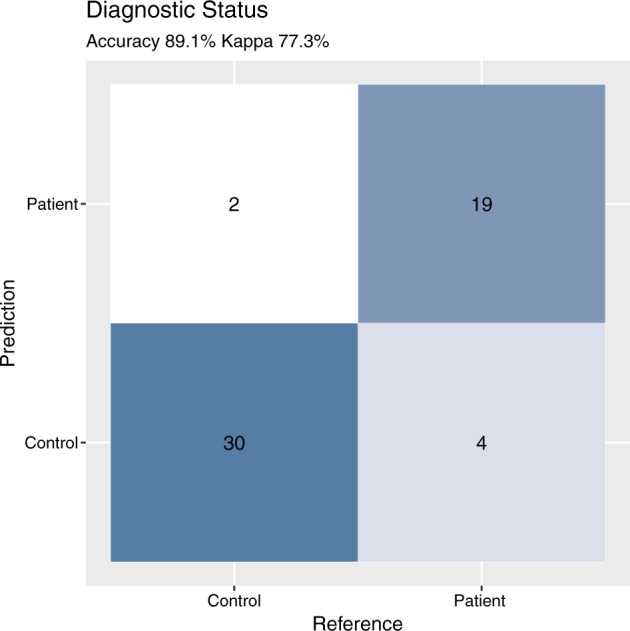
This plot depicts the predictions the confusion matrix between the predicted group (i.e., control or patient) and the observed reference group

**Fig. 2:**
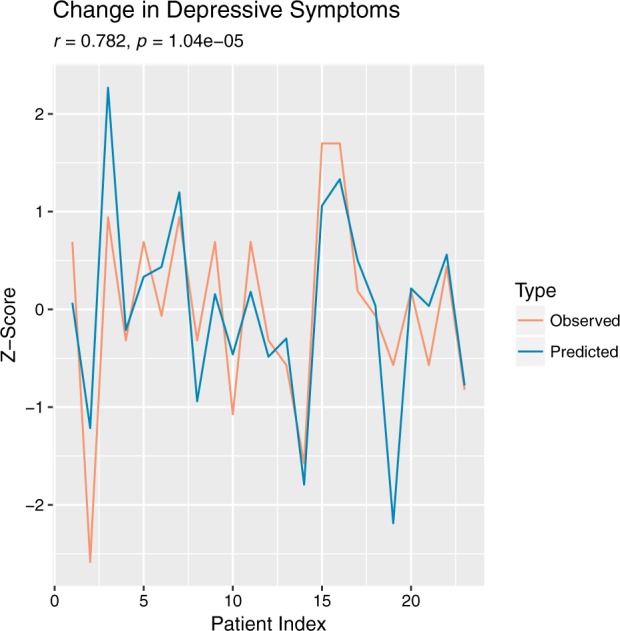
This plot depicts the predicted and actual pre–post difference scores in depressive symptoms that were sample-standardized for visualization for each of the 23 patients

Using actigraphy features alone, the machine-learning algorithm correctly predicted the diagnostic status 89% of the time (*p* = 5.5e-07), with a corresponding Cohen’s kappa of 0.773, sensitivity of 0.937, and specificity of 0.826 (see Fig. 1). The correlation between predicted and actual change in depression severity was strong (*r* = 0.782, *p* = 1.04e-05; see Fig. 2).

## Discussion

The present study aimed to identify digital biomarkers of MDD and bipolar disorder. Up to 2 weeks of actigraphy data distinguished patients from healthy controls with high accuracy. The current approach substantially improved upon earlier results,^4^ and it classified diagnostic status more accurately than published inter-reliability rates for second raters using the SCID-I/P.^10^ Results suggest that unobtrusive, passive actigraphy data hold potential to supplement traditional diagnostic assessments of MDD and bipolar disorder. By reducing reliance on resource-intensive assessment approaches in favor of low-burden, low-cost digital data, it may be possible to detect MDD and bipolar disorder earlier and more broadly.

Results also showed that actigraphy data predicted the majority of variation in patients’ depression severity over ~2 weeks. Whereas most digital biomarker research has focused on diagnostics,^3^ use of passive monitoring to detect symptom change may enable just-in-time interventions as a next step. For example, temporally sensitive detection of clinical deterioration via passive sensors could be used to alert the patient and suggest use of therapeutic skills (e.g., behavioral activation), as well as to alert the clinician, prompting timely clinical intervention.

In addition to the strong predictive ability of our model, another strength of the present approach was its use of features that are generalizable, so that methods may be replicated in new samples. The field is in early stages of using passive data to enhance diagnostics. As such, this early study should also be interpreted bearing in mind its limitations. We elected to examine MDD and bipolar disorder together, rather than dividing them into smaller groups, to enhance power. On one hand, our ability to differentiate one’s broad mood disorder status from healthy controls points to detection of potential transdiagnostic characteristics, enhancing the generalizability of results. Alternatively, predicting patients with MDD separately from those with bipolar disorder may enhance detection accuracy further, as a next step in a larger sample. Additionally, because the public-use dataset did not report participants’ medication data or other treatment received, we were unable to assess how study movement might have been related to medication or psychotherapy. Further, five patients were in an inpatient facility during data collection, which likely constricted their movement. Finally, diagnostic status was established at baseline; it is possible that prospective studies designed to predict later diagnostic status would yield different results. Although chances of overfitting are reduced using LOOCV and boosting models,^11^ current results are preliminary and require replication by independent research groups, in larger samples. If replicated, results suggest that passive digital assessment show promise for reducing the burden of MDD and bipolar disorder.

## Methods

### Participants

Twenty-three patients (22% inpatient, 78% outpatient, *M*_*Age*_ = 42.8, *SD*_*Age*_ = 11.0, 65% with primary MDD, 30% with primary bipolar II, and 4% with primary bipolar I, 57% male, 13% currently working) were invited to participate by study staff when they came for clinical care in the medical setting and 32 controls (*M*_*Age*_ = 38.2, *SD*_*Age*_ = 13.0, 37.5% male) without a history of mood or psychotic symptoms were invited to participate by study staff from hospital employee, university, or primary care settings.^5^ Fifteen patients received antidepressants, and some were co-medicated with lithium, mood stabilizers, antipsychotics, anxiolytics, or hypnotics. Eight patients did not use psychotropic medications. All participants were consented and the study received local ethics committee approval (REK III, Health -West, Norway).^6^ Data were collected from May 2002 through February 2006.

### Clinical assessment

Participants were assessed for mood disorder diagnosis by a psychiatrist using the Structured Clinical Interview for DSM-IV (SCID-I).^6^ The psychiatrist also administered the Montgomery-Asberg Depression Scale (MADRS) to patients, to assess depressive symptoms before and after the actigraphy study.^6^ Change was evaluated using pre-post difference scores in depressive symptoms and were then sample-standardized for graphical visualization.

### Actigraphy

Participants wore an actigraph on their right wrist (actiwatch) for up to 2 weeks, to continuously monitor movement. Actigraphs were worn at all times, except when bathing. The sampling frequency was 32 Hz and movements of ≥0.05 g were recorded. Voltage of movement was recorded for each minute. A timeframe of 2 weeks was selected balancing device battery life and amount of time considered sufficient, as an early study on motor activation.^5^

### Planned analyses

Prior to analyses, features were extracted from actigraphy data to form a set of generalizable digital biomarkers. Digital biomarkers included person-level measures of (1) movement intensity distribution (i.e., minimum, maximum, mean, median, modal, skewness, kurtosis, and 1st–99th quantiles), (2) movement intensity variability (i.e., root mean-square of successive differences [RMSSD] between movement 1 to 2 min later, which is an index of sharp shifts in movement across short time intervals), and the standard deviation of movement), (3) autoregressive lags of movement intensity (i.e., consistency of movement 1 to 100 min later across a smooth continuum using the differential time-varying effect model),^7^ and (4) oscillatory pattern (i.e., spectral densities of the movement patterns), resulting in a total of 9929 features. Features were not reliant on absolute time in the study, but rather used relative time differences among patterns in the data. Biomarkers were created based on the minimum number of minutes of actigraphy data that were available for all participants (i.e., all participants had 19,299 min of actigraphy data), and consequently, there was no missing data in any derived biomarkers.

Results were analyzed using extreme gradient boosting (xgboost), which is a tree-based boosting system^8^ that offers high precision.^9^ Diagnostic group membership (i.e., patient, control) and change in depressive symptoms were predicted from the digital biomarkers. Model regularization methods, leave-one-out cross-validation (LOOCV), and permutation tests controlled for overfitting. Primary outcome measures were percentage of diagnostic agreement, sensitivity, specificity, and Cohen’s kappa when predicting diagnostic group membership, and the correlation between predicted and actual values for change in depressive symptoms (i.e., the models predicted pre-post difference scores, then standardized to sample z-scores for visualization).

### Reporting summary

Further information on research design is available in the Nature Research Reporting Summary.

## Supplementary information

Nature Research Reporting Summary Checklist

## Data Availability

Data and scripts used to reproduce results are available at http://www.nicholasjacobson.com/post/digital_biomarkers_mood_disorders/.

## Supplementary information

**Supplementary information** accompanies the paper on the *npj Digital Medicine* website (10.1038/s41746-019-0078-0).
