# Supplementary material for: Digital biomarkers of mood disorders and symptom change
Source: NPJ Digit Med. 2019 Feb 1;2:3. doi: 10.1038/s41746-019-0078-0 (PMC6550284; doi:10.1038/s41746-019-0078-0)
Supplement: Supplementary file 1 — Nature Research Reporting Summary Checklist [file 41746_2019_78_MOESM1_ESM.pdf]

nature research

Corresponding author(s): Nicholas Jacobson

Last updated by author(s): Jan 8, 2019

Reporting Summary

Nature Research wishes to improve the reproducibility of the work that we publish. This form provides structure for consistency and transparency in reporting. For further information on Nature Research policies, see Authors & Referees and the Editorial Policy Checklist.

Statistics

For all statistical analyses, confirm that the following items are present in the figure legend, table legend, main text, or Methods section.

n/a	Confirmed	
☐	☒	The exact sample size (n) for each experimental group/condition, given as a discrete number and unit of measurement
☐	☒	A statement on whether measurements were taken from distinct samples or whether the same sample was measured repeatedly
☐	☒	The statistical test(s) used AND whether they are one- or two-sided<br>*Only common tests should be described solely by name; describe more complex techniques in the Methods section.*
☐	☒	A description of all covariates tested
☐	☒	A description of any assumptions or corrections, such as tests of normality and adjustment for multiple comparisons
☐	☒	A full description of the statistical parameters including central tendency (e.g. means) or other basic estimates (e.g. regression coefficient) AND variation (e.g. standard deviation) or associated estimates of uncertainty (e.g. confidence intervals)
☐	☒	For null hypothesis testing, the test statistic (e.g. F, t, r) with confidence intervals, effect sizes, degrees of freedom and P value noted<br>*Give P values as exact values whenever suitable.*
☒	☐	For Bayesian analysis, information on the choice of priors and Markov chain Monte Carlo settings
☒	☐	For hierarchical and complex designs, identification of the appropriate level for tests and full reporting of outcomes
☐	☒	Estimates of effect sizes (e.g. Cohen's d, Pearson's r), indicating how they were calculated

Our web collection on statistics for biologists contains articles on many of the points above.

Software and code

Policy information about availability of computer code

Data collection	Data was based on public use data.
Data analysis	Models were analyzed with R 3.5.1

For manuscripts utilizing custom algorithms or software that are central to the research but not yet described in published literature, software must be made available to editors/reviewers. We strongly encourage code deposition in a community repository (e.g. GitHub). See the Nature Research guidelines for submitting code & software for further information.

Data

Policy information about availability of data

All manuscripts must include a data availability statement. This statement should provide the following information, where applicable:

- Accession codes, unique identifiers, or web links for publicly available datasets
- A list of figures that have associated raw data
- A description of any restrictions on data availability

Data and scripts used to reproduce results are available at http://www.nicholasjacobson.com/post/digital_biomarkers_mood_disorders/.

Field-specific reporting

Please select the one below that is the best fit for your research. If you are not sure, read the appropriate sections before making your selection.

☐ Life sciences ☒ Behavioural & social sciences ☐ Ecological, evolutionary & environmental sciences

For a reference copy of the document with all sections, see nature.com/documents/nr-reporting-summary-flat.pdf

Behavioural & social sciences study design

All studies must disclose on these points even when the disclosure is negative.

Study description	The current study involved a secondary analysis of public use actigraphy data from wearable sensors using machine learning to predict diagnosis and symptom change.
Research sample	Twenty-three patients (22% inpatient, 78% outpatient, MAge=42.8, SDAge=11.0, 65% with primary MDD, 30% with primary bipolar II, and 4% with primary bipolar I, 57% male, 13% currently working) were invited to participate by study staff when they came for clinical care in the medical setting and 32 controls (MAge=38.2, SDAge=13.0, 37.5% male) without a history of mood or psychotic symptoms were invited to participate by study staff from hospital employee, university, or primary care settings.
Sampling strategy	The current sample was based on a public-use data collected using a convenience sample.
Data collection	Clinical Assessment<br>Participants were assessed for mood disorder diagnosis by a psychiatrist using the Structured Clinical Interview for DSM-IV (SCID-I).6 The psychiatrist also administered the Montgomery-Asberg Depression Scale (MADRS) to patients, to assess depressive symptoms before and after the actigraphy study.6 Change was evaluated using pre-post differences scores in depressive symptoms and were then sample-standardized for graphical visualization.<br><br>Actigraphy<br>Participants wore an actigraph on their right wrist (Actiwatch) for up to 2 weeks, to continuously monitor movement. Actigraphs were worn at all times, except when bathing. The sampling frequency was 32 Hz and movements of > 0.05g were recorded. Voltage of movement was recorded for each minute. A timeframe of 2 weeks was selected balancing device battery life and amount of time considered sufficient, as an early study on motor activation.
Timing	Data were collected from May 2002 through February 2006.
Data exclusions	N/A
Non-participation	As this was based on a public-use dataset, this was unavailable.
Randomization	N/A

Reporting for specific materials, systems and methods

We require information from authors about some types of materials, experimental systems and methods used in many studies. Here, indicate whether each material, system or method listed is relevant to your study. If you are not sure if a list item applies to your research, read the appropriate section before selecting a response.

Materials & experimental systems

n/a	Involved in the study	
☒	☐	Antibodies
☒	☐	Eukaryotic cell lines
☒	☐	Palaeontology
☒	☐	Animals and other organisms
☐	☒	Human research participants
☐	☒	Clinical data

Methods

n/a	Involved in the study	
☒	☐	ChIP-seq
☒	☐	Flow cytometry
☒	☐	MRI-based neuroimaging

Human research participants

Policy information about studies involving human research participants

Population characteristics	See above.
Recruitment	Participants were recruited using a convenience sample (see above).
Ethics oversight	The study was approved by an ethics committee (REK III, Health -West, Norway).

Note that full information on the approval of the study protocol must also be provided in the manuscript.

Clinical data

Policy information about clinical studies

All manuscripts should comply with the ICMJE guidelines for publication of clinical research and a completed CONSORT checklist must be included with all submissions.

Clinical trial registration	N/A
Study protocol	The current study is based on secondary analysis of public-use data, and consequently was not pre-registered.
Data collection	See above.
Outcomes	For the present study the primary outcomes were the diagnostic status of participants and the change in depressive symptoms.
